# Epigenetic Clocks, Resilience, and Multi-Omics Ageing: A Review and the EpiAge-R Conceptual Framework

**DOI:** 10.3390/ijms27041908

**Published:** 2026-02-17

**Authors:** Hidekazu Yamada

**Affiliations:** Anti-Aging Center, Kindai University, 1-14-1 Miharadai, Minami-ku, Sakai City 590-0197, Osaka, Japan; yamadahi@med.kindai.ac.jp; Tel.: +81-90-8799-5590

**Keywords:** epigenetic clocks, biological resilience, ageing biomarkers, multi-omics integration, nanopore sequencing, health capital, flourishing, aging clocks

## Abstract

Epigenetic clocks have successfully estimated biological age by identifying CpG sites whose DNA methylation levels correlate with chronological age. However, these statistical models provide limited mechanistic insight into the biological underpinnings of ageing. While they capture the “pace” of ageing, they fail to quantify the “resilience” of biological systems—the capacity to recover, reorganize, and maintain homeostasis under stress. To overcome this limitation, we introduce EpiAge-R (Epigenetic Age with Resilience), a mechanistic framework that shifts the focus from passive correlation to active recovery potential. The EpiAge-R framework integrates multilayered biological information—including long-read methylation sequencing, chromatin context, histone modification balance, 3D genome topology, and mitochondrial dynamics—into a unified Resilience Index. By distinguishing between degenerative methylation drift (damage) and adaptive repair processes (resilience), EpiAge-R aligns with nonlinear multi-omics ageing trajectories. This framework provides a quantitative foundation for next-generation biomarkers and precision longevity interventions, aiming to define optimal health rather than statistical normality.

## 1. Introduction

DNA methylation clocks, such as the seminal Horvath and Hannum models, have revolutionized geroscience by providing highly accurate estimators of chronological age [1,2]. While later generations of clocks—including GrimAge and DunedinPACE—have improved predictive power for mortality and the pace of ageing [3,4], a critical limitation remains: these statistical models are largely correlational. They function as odometers or speedometers, capturing the accumulated damage or the rate of decline, but they fail to quantify the biological system’s resilience—the active capacity to recover, reorganize, and maintain homeostasis after stress [5,6].

Ageing is increasingly understood not merely as a linear accumulation of deficits, but as a dynamic struggle between damage (entropy) and repair mechanisms [7,8]. Current methylation clocks, however, cannot distinguish between degenerative methylation drift and adaptive compensatory changes [9]. Consequently, two individuals with the same “biological age” may possess vastly different capacities to rebound from a stressor, such as surgery or infection.

To bridge this gap, we propose EpiAge-R (Epigenetic Age with Resilience), a mechanistic framework designed to quantify this missing dimension [10]. Unlike traditional black-box predictors, EpiAge-R integrates multi-omics layers—ranging from chromatin topology and histone modifications to physiological resilience metrics—to separate the signal of “damage” from the potential for “repair.” In this Review, we delineate the limitations of current correlational clocks and introduce the conceptual architecture of EpiAge-R, providing a roadmap for next-generation biomarkers that guide precision longevity interventions.

## 2. Conceptual Framework from Damage to Resilience 

**Figure 1:**
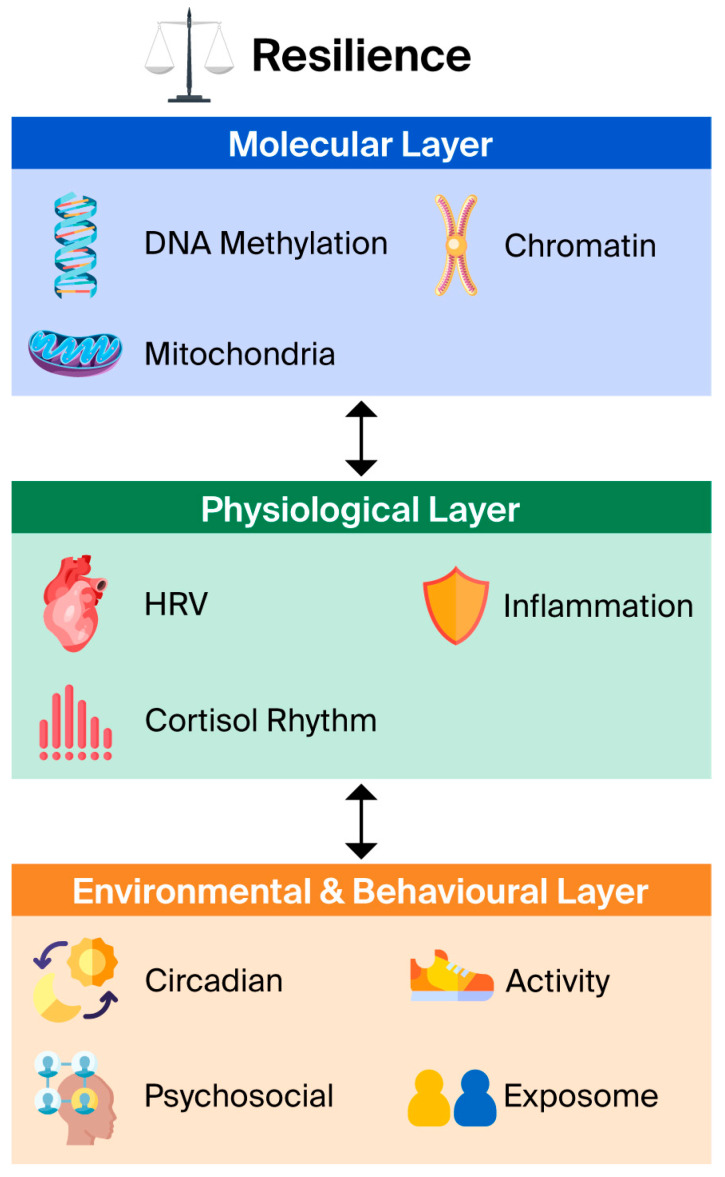
**The hierarchical architecture of the EpiAge-R framework.** The model consists of three interacting layers that collectively determine resilience. (1) **Molecular Layer:** DNA methylation, chromatin topology, and mitochondrial function. (2) **Physiological Layer**: Systemic metrics including heart-rate variability (HRV), inflammation, and cortisol rhythms. (3) **Environmental & Behavioral Layer:** Circadian alignment, physical activity, and psychosocial factors. Bidirectional arrows represent the dynamic feedback loops between layers.

Ageing arises as a system-level phenomenon driven by cumulative molecular damage and the progressive decline of repair capacity [5]. This conceptual framework synthesizes existing multi-omics evidence and does not present new experimental data. To capture this complexity, EpiAge-R is structured around three interacting layers—molecular, physiological, and environmental—that together quantify an individual’s biological resilience [10] (Figure 1).

### 2.1. Limitations of Correlational CpG-Based Clocks

Conventional epigenetic clocks are statistical estimators designed to predict chronological age from panels of CpG sites. While highly accurate as predictive tools, they provide limited mechanistic insight because the selected CpGs vary across datasets, tissues, and algorithms, reflecting population-level correlations rather than conserved biological processes [9]. Specifically, these models face challenges with tissue heterogeneity, often struggling to resolve cell-type specific ageing patterns without extensive deconvolution [11]. Furthermore, they typically assume a linear progression of methylation changes, whereas identifying novel loci has revealed non-linear relationships associated with physiological shifts such as menarche or metabolic changes [12]. Most critically, correlational clocks rarely identify upstream drivers—such as chromatin structure, histone balance, or mitochondrial dysfunction—that causally govern the ageing process [8,13,14].

### 2.2. The EpiAge-R Approach

**Figure 2:**
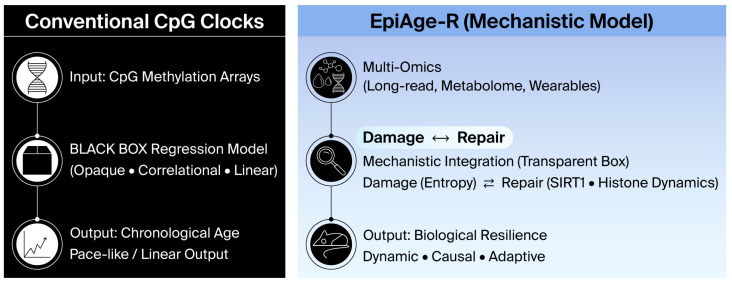
**Paradigm shift from correlational to mechanistic ageing clocks.** (**Left**) Conventional CpG clocks function as a “black box,” using linear regression to predict chronological age based on correlation. They typically output a linear pace of ageing. (**Right**) EpiAge-R employs a “transparent box” approach, integrating multi-omics data to model the dynamic equilibrium between **Damage (Noise)** and **Repair (Resilience)**. This mechanistic integration outputs a score of Biological Resilience, distinguishing actionable biological drivers from passive decay.

EpiAge-R moves beyond CpG prediction by focusing on the damage–repair pathways that determine epigenomic plasticity [15]. It conceptualizes ageing as a loss of resilience. This framework draws inspiration from cross-system multi-omics profiling, which highlights dysregulation across organ networks [16,17,18]. By integrating emerging technologies such as nanopore sequencing, EpiAge-R characterizes how ageing progresses—not merely how far—thereby enabling quantitative assessment of resilience [19,20] (Figure 2).

### 2.3. Comparative Analysis: Pace, State, and Resilience

To position EpiAge-R within the current landscape of geroscience, it is essential to distinguish it from established biomarkers. Second-generation clocks, such as GrimAge and PhenoAge, were trained against mortality and morbidity, making them powerful predictors of “biological state” and remaining lifespan [3]. Third-generation metrics, most notably DunedinPACE, measure the “pace of ageing”—the instantaneous rate of biological decline.

However, a critical gap remains: neither “state” nor “pace” metrics explicitly quantify the system’s capacity to rebound from stressors. For example, two individuals may share the same GrimAge (biological age) and DunedinPACE (rate of decline), yet exhibit vastly different outcomes following a hip fracture or viral infection. This divergence is determined by resilience. EpiAge-R addresses this specific dimension. While GrimAge acts as an odometer (distance travelled) and DunedinPACE as a speedometer (current velocity), EpiAge-R functions as a “shock absorber” gauge. By integrating the Repair Capacity Index (RCI) and Physiological Resilience Index (PRI), it identifies individuals who, despite having an accelerated “pace” of ageing due to transient stress, possess the molecular reserve to decelerate and repair—a distinction crucial for evaluating the efficacy of rejuvenation therapies.

## 3. The Molecular Layer: Epigenetic and Chromatin Resilience

At the molecular level, EpiAge-R integrates information from DNA methylation, histone modifications, chromatin topology, telomere maintenance, and mitochondrial function to define the “Epigenetic Damage Index” (EDI) and “Repair Capacity Index” (RCI) [10].

### 3.1. Nanopore Sequencing and Chromatin Context

Nanopore long-read sequencing enables the concurrent measurement of DNA methylation and surrounding chromatin context, preserving spatial and structural information that short-read arrays typically lose [20,21]. Accurate mapping using advanced aligners ensures robust detection of methylation states even in repetitive regions [22]. This approach reveals nucleosome phasing, histone occupancy, and higher-order interactions [19]. EpiAge-R leverages these data to define a chromatin-resolved methylome, interpreting methylation variability with respect to chromatin accessibility. Crucially, this method allows for the distinction between stable methylation maintained by DNA methyltransferases (DNMT1, DNMT3A/3B) and active demethylation intermediates (e.g., 5-hydroxymethylcytosine) generated by Ten-Eleven Translocation (TET) enzymes, providing a dynamic view of epigenomic plasticity. Patterns of methylation discordance (Shannon entropy) across long reads serve as indicators of chromatin instability—a hallmark of ageing [21].

### 3.2. Histone-Modification Balance

The balance between activation and repression is critical for resilience. The H3K27ac/H3K9me3 ratio reflects this activation-to-repression balance. This balance is dynamically regulated by the opposing actions of histone acetyltransferases (HATs) and histone deacetylases (HDACs), as well as methyltransferases like EZH2, which catalyzes the formation of the repressive H3K27me3 mark. Ageing shifts toward repressive marks, reducing chromatin plasticity [13]. Enrichment of H3K27ac at repair genes indicates adaptive capacity, while its loss signals senescence [23].

### 3.3. Nuclear Mechanobiology and 3D Genome Architecture

Epigenetic resilience is not governed solely by chemical modifications but is physically constrained by nuclear mechanobiology. The nucleus serves as a mechanosensor, integrating physical forces from the microenvironment via the LINC complex (Linker of Nucleoskeleton and Cytoskeleton). In ageing tissues, particularly dermal fibroblasts, the degradation of the extracellular matrix (ECM) leads to a loss of cytoskeletal tension. This mechanical decoupling triggers the downregulation of Lamin A/C, a key structural protein that anchors heterochromatin to the nuclear periphery. The consequent softening of the nucleus results in the detachment of Lamina-Associated Domains (LADs) and the decompaction of constitutive heterochromatin [24,25].

This “mechanical ageing” directly exposes previously silenced transposons (e.g., LINE-1) to transcriptional machinery. Crucially, the leakage of derepressed retrotransposable elements into the cytoplasm triggers the cGAS-STING innate immune pathway [26]. This “viral mimicry” mechanism serves as the molecular bridge converting nuclear structural damage (EDI) into the systemic inflammation (PRI) observed in the physiological layer. Thus, EpiAge-R explicitly incorporates “Nuclear Stiffness” and “LAD Integrity” to capture this physical dimension of resilience.

### 3.4. Telomere Integrity and Mitochondrial Epigenetics

Beyond mean length, EpiAge-R integrates telomere-length distribution and subtelomeric methylation, quantified via high-throughput qPCR or sequencing methods [27,28]. Hypomethylation of subtelomeres exposes ends to ROS-mediated injury, while heterogeneity indicates high turnover risk [28,29]. Furthermore, mitochondrial dysfunction propagates epigenetic drift. EpiAge-R integrates mtDNA copy number and methylation as molecular correlates of energy resilience [30,31].

## 4. The Physiological Layer: Systemic Adaptability

The physiological layer captures intermediate phenotypes reflecting the body’s buffering capacity against environmental and metabolic perturbations [32]. Recent advances have expanded this layer to include multi-organ and metabolomic clocks [33,34,35,36].

### 4.1. Autonomic and Neuro-Endocrine Resilience 

A primary component of physiological resilience is autonomic regulation, measured via post-stress Heart-Rate Variability (HRV). Low variability indicates autonomic imbalance and is a strong predictor of frailty [32], a gap that wearable-based clocks can now monitor continuously in daily life [37]. This autonomic interplay extends to endocrine flexibility, specifically the diurnal cortisol gradient and the DHEA-S/cortisol ratio. A flattened cortisol slope is associated with chronic stress and cognitive decline [29,38], aligning with findings that epigenetic acceleration predicts midlife cognitive function [39].

### 4.2. Systemic Integrity and Inflammation 

At the systemic level, new “Systems Age” metrics and metabolomic clocks quantify ageing heterogeneity across organs (e.g., renal, hepatic) [33,36]. Intrinsic capacity clocks further refine this by predicting mortality based on functional reserves [35]. Crucially, these systems are modulated by inflammation–resolution dynamics, specifically the ratio of IL-6 to IL-10. Robust dynamics signify resolution capacity, whereas chronic elevation predicts cardiovascular risk [40]. This is paralleled by immune diversity, where reduced T-cell-receptor (TCR) repertoire entropy correlates with immunosenescence and reduced adaptability [41]. Finally, neurological resilience metrics derived from brain-specific multi-omics reveal mechanisms of proteostatic collapse, distinguishing healthy brain ageing from pathological trajectories [42,43].

## 5. The Environmental Layer: Adaptive Context

The environmental layer integrates exosomal and behavioural adaptation, recognising that genetic factors alone cannot explain chronic disease risks [44]. This “exposome” concept complements the genome by measuring lifetime environmental exposures [45], captured in the Behavioural/Environmental Index (BAI).

### 5.1. Behavioural Modulation and Social Genomics 

Modifiable behavioural drivers play a central role in resilience. Circadian alignment—the synchrony between epigenetic rhythmicity and cortisol/melatonin cycles—is critical, as misalignment accelerates clocks via oxidative stress [8,29]. Similarly, physical activity enhances mitochondrial biogenesis and metabolic flexibility through the AMPK-SIRT1 signaling pathway, countering age-related bioenergetic decline [46]. Psychosocial engagement also acts as a biological input; methylation of OXTR and BDNF genes serves as a molecular interface between the social environment and neural function. Mechanistically, social connectedness mitigates chronic HPA-axis hyperactivation, preventing the glucocorticoid-resistance that drives systemic inflammation. Conversely, isolation and trauma induce epigenetic silencing of glucocorticoid response elements, accelerating the inflammatory ageing clock [47,48,49].

### 5.2. Exogenous Stressors 

These behavioural factors interact with exogenous stressors. Exposure to pollutants (e.g., PM2.5) and early-life environmental adversities significantly accelerate epigenetic ageing [50,51,52]. These environmental states are often transmitted across tissues via extracellular vesicles (EVs), which carry stress or repair signals [53].

## 6. Integrative Scoring: The EpiAge-R Index

### 6.1. The Resilience Index Formula

**Figure 3:**
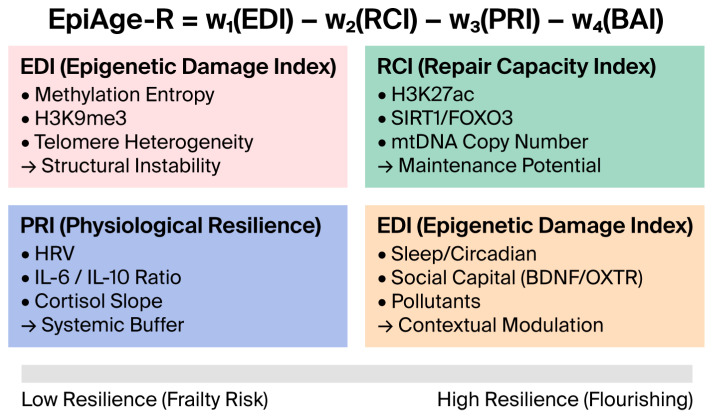
Composition of the EpiAge-R Index.

The EpiAge-R score is calculated as follows: EpiAge-R = w1(EDI) − w2(RCI) − w3(PRI) − w4(BAI) (Figure 3).

The components are defined as follows. The Epigenetic Damage Index (EDI) quantifies structural instability by aggregating methylation entropy, repressive histone marks, and telomere heterogeneity [14,21,28]. Counterbalancing this is the Repair Capacity Index (RCI), which reflects maintenance potential based on histone activation (H3K27ac), mtDNA copy number, and longevity gene activity (e.g., SIRT1/FOXO3) [8,30,54]. At the systemic level, the Physiological Resilience Index (PRI) captures buffering capacity through HRV, cortisol slopes, and inflammation resolution ratios [32,33,40]. Finally, the Behavioural/Environmental Index (BAI) accounts for contextual modulation, weighting factors such as circadian alignment, social capital (BDNF/OXTR), and pollutant burden [45,47].

Weighting factors (w1–w4) are optimized using machine learning approaches such as Elastic Net [55] or Random Forest [56], and potentially Deep Learning frameworks [57,58]. Crucially, unlike traditional clocks, these weights must be calibrated against phenotypic “ground truths” of resilience, such as recovery rates following standardized stressors or frailty indices, ensuring the model captures active recovery capacity rather than passive time.

The total resilience score is calculated by subtracting damage from resilience capacities:

EpiAge-R = w1(EDI) − w2(RCI) − w3(PRI) − w4(BAI).

- **EDI (Epigenetic Damage Index)**: Quantifies structural instability (e.g., methylation entropy).
- **RCI (Repair Capacity Index)**: Reflects maintenance potential (e.g., SIRT1 activity).
- **PRI (Physiological Resilience Index)**: Captures systemic buffering (e.g., HRV).
- **BAI (Behavioral/Environmental Index)**: Represents contextual adaptation.

The bar at the bottom illustrates the continuum from Low Resilience (Frailty Risk) to High Resilience (Flourishing).

**Step-wise Implementation Strategy:** While the full EpiAge-R model integrates all four domains for maximum precision, we propose a step-wise implementation strategy to ensure clinical feasibility. A simplified “Core EpiAge-R”, utilizing only the molecular components (EDI and RCI), could be deployed immediately using blood-based nanopore sequencing. Even without physiological or environmental data, this core model offers a significant advantage over traditional clocks by mechanistically distinguishing between accumulated damage (entropy) and repair potential (histone/enzymatic capacity). Subsequent layers (PRI and BAI) can be integrated as “modules” depending on data availability and cost constraints, allowing the framework to scale from basic research to comprehensive clinical monitoring.

### 6.2. The Flourishing Extension

For analysis of positive health assets, the model extends to Epi-Flourishing [47,48]: Epi-Flourishing = Resilience Index + Asset Factor where the Asset Factor includes psychosocial and neuroplasticity proxies (e.g., BDNF demethylation, social capital) and subjective well-being indices [47,59]. Cumulative social advantage has been linked to slower epigenetic ageing, supporting this asset-based view [48]. This quantification tracks how lifestyle interventions foster a regenerative paradigm in which accumulated health assets drive sustained healthspan [46,47]. This formulation is intended as a conceptual scoring scheme rather than a validated clinical tool. Future empirical studies will be required to calibrate the weighting factors and assess the robustness of the EpiAge-R score across cohorts and tissues.

### 6.3. Operationalization: A Minimum Viable Protocol

To transition EpiAge-R from a conceptual framework to an analytical tool, we propose a “minimum viable score” (MVS) protocol using currently accessible biomarkers.

#### 6.3.1. Feature Selection

First, the Epigenetic Damage Index (EDI) is calculated using Shannon entropy across cytosines covered by long-read sequencing (e.g., Oxford Nanopore), focusing on regions with high cell-to-cell variability (epigenetic noise). Second, the Repair Capacity Index (RCI) is derived as a composite score of mtDNA copy number (qPCR) and the expression levels of key longevity genes (e.g., SIRT1, FOXO3), normalized against chronological age. Third, the Physiological Resilience Index (PRI) incorporates heart-rate variability (SDNN) and IL-6/IL-10 ratios derived from standard blood panels.

#### 6.3.2. Normalization and Integration

Raw values for each layer are converted to Z-scores (Z = (x − μ)/σ) based on age- and sex-matched reference populations to ensure comparability across different units. The modular design allows for partial scoring. The “Core EpiAge-R” (EDI/RCI only) offers high molecular resolution and accessibility, requiring only blood samples, though it may lack predictive accuracy for systemic outcomes compared to the “Whole EpiAge-R.” While the Core model captures cellular potential, the inclusion of physiological (PRI) and environmental (BAI) layers in the Whole model serves as a “contextual multiplier,” which is essential for predicting real-world recovery trajectories such as post-surgical rehabilitation.

## 7. Discussion and Clinical Implications

In this Review, we synthesised recent advances in epigenetic clocks, multi-omics ageing, and resilience biology, and integrated them into the EpiAge-R conceptual framework.

### 7.1. From Damage to Resilience

**Figure 4:**
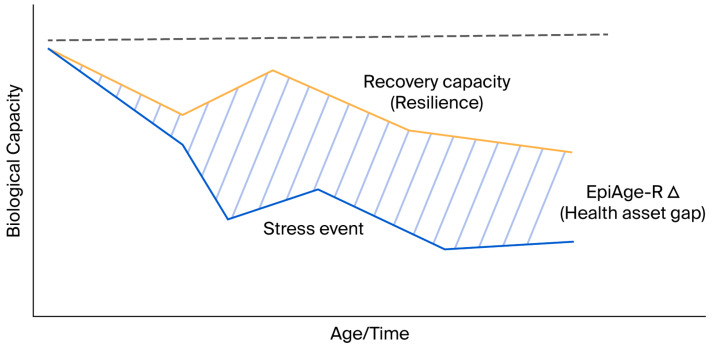
**Conceptual trajectory of biological resilience.** The graph illustrates biological capacity (*y*-axis) over time (*x*-axis). Upon encountering a **Stress Event,** individuals with high resilience (orange line) exhibit robust **Recovery Capacity,** returning to baseline homeostasis. In contrast, low resilience (blueline) leads to incomplete recovery and an increased risk of frailty. The **EpiAge-R Δ (Health Asset Gap)** quantifies the functional reserve differentiating a flourishing trajectory from a frailty trajectory.

The EpiAge-R framework provides a new conceptual lens for understanding biological ageing—not as a linear accumulation of molecular damage, but as a progressive modulation of resilience [5,38]. Traditional molecular markers describe deterioration yet fail to capture the intrinsic recovery capacity. By quantifying resilience, EpiAge-R allows for the assessment of both degeneration and rejuvenation potential [60]. Epigenetic reprogramming studies demonstrate that ageing is reversible [61]. For example, partial reprogramming and interventions with FOXO3-enhanced stem-cell-derived progenitors have been shown to reverse epigenetic clocks; EpiAge-R captures these dynamics via the Repair Capacity Index (RCI) [54,62] (Figure 4).

### 7.2. Addressing the Challenge of Biological Validation and Normativity Bias

A major critique of current epigenetic clocks is their “black box” nature—they predict outcomes with high accuracy but low interpretability. Recently, the **Biomarkers of Aging Consortium**, led by Moqri et al., proposed a standardized framework for classifying biomarkers into predictive, responsive, and biological categories [63]. They emphasize that for a biomarker to be actionable, it must satisfy “**biological validation**” criteria: it should elucidate the causal mechanisms driving the aging process rather than merely correlating with time.

Furthermore, in their recent consensus regarding “what makes clocks tick,” the Consortium highlights the need to disentangle the distinct molecular components of ageing [64]. EpiAge-R is directly responsive to this call. By explicitly separating “Damage” (entropy, instability) from “Repair” (enzymatic capacity), EpiAge-R provides a transparent architecture that allows researchers to pinpoint **why** an individual’s biological age is accelerated.

Critically, current clinical interpretations often view a biological age that matches chronological age as “normal” or “healthy.” However, EpiAge-R challenges this “normativity bias.” Recent discourse in the longevity community argues that “normal ageing” inherently entails progressive accumulation and metabolic decline. Therefore, the goal of next-generation biomarkers should not be to validate “standard” decline (i.e., being average for one’s age), but to quantify the “deviation into resilience”—the capacity to remain functionally younger and more robust than the chronological norm. This shift aligns with the emerging consensus that ageing itself is a treatable condition, requiring metrics that define optimal health rather than statistical normality.

### 7.3. Generative AI and Multi-Omics Integration

The integration of the four layers of EpiAge-R (Molecular to Environmental) generates a high-dimensional dataset that exceeds the capacity of linear regression models. To optimize the weighting factors (w1–w4) and resolve non-linear interactions, the application of Generative AI and Transformer-based models is indispensable. Recent work by Zhavoronkov and colleagues has demonstrated the utility of generative AI platforms **(e.g., PandaOmics)** in analyzing longitudinal multi-omics data to identify “dual-purpose targets”—pathways that simultaneously modulate ageing and disease processes. By leveraging these deep learning frameworks, EpiAge-R can evolve from a static score into a generative simulation tool [65]. For instance, “in silico” perturbation analysis could predict how specific lifestyle modifications would quantitatively improve an individual’s Resilience Index. This convergence of mechanistic biology and generative AI represents the next frontier in precision longevity medicine.

### 7.4. Navigating Genomic Privacy via Epigenetic Inference

While epigenetics is central to plasticity, the foundational role of genetics cannot be ignored. Classic models like the **Agouti mouse** demonstrate that Single Nucleotide Polymorphisms (SNPs) fundamentally dictate phenotypic potential [66]. In humans, specific functional SNPs drive a significant portion of protein expression variability. However, direct sequencing of germline DNA faces increasing regulatory hurdles globally due to privacy concerns (e.g., GDPR), limiting its widespread use [67].

Here, the EpiAge-R framework offers a strategic breakthrough. Since functional SNPs often manifest their effects through **allele-specific methylation (ASM)** and chromatin remodeling (methylation Quantitative Trait Loci, or **mQTLs**) [68], epigenetic patterns can serve as a functional proxy for genetic risk. By employing AI algorithms trained on multi-omics data, it becomes possible to infer the “functional output” of high-risk SNPs solely from epigenetic and proteomic data, without directly sequencing the germline genome. This approach shifts the paradigm from analyzing “immutable personal data” to monitoring “dynamic biological states,” aligning with stricter data privacy regulations.

### 7.5. Biological Integration of Psychosocial Assets

Recent advances in social genomics have demonstrated that psychological states correlate with distinct molecular signatures. Specifically, research distinguishing between hedonic well-being and eudaimonic well-being (sense of purpose) reveals that eudaimonia is associated with the downregulation of the Conserved Transcriptional Response to Adversity (CTRA)—a gene expression profile characterized by pro-inflammatory signaling and reduced antiviral responses [69]. EpiAge-R operationalises these findings by treating psychosocial assets not as abstract concepts, but as measurable inputs (e.g., BDNF methylation, CTRA gene regulation) within the Asset Factor. This approach grounds the “flourishing” component in established molecular biology, proposing that subjective well-being actively modulates biological resilience via neuro-endocrine-immune pathways.

### 7.6. Clinical Translation and Therapeutic Interventions

EpiAge-R supports precision-longevity interventions by acting as a dynamic efficacy monitor. It guides personalised evaluation of therapeutic responses to interventions such as mitochondrial activation and epigenetic reprogramming [53]. Newer benchmarking tools like LinAge2 provide actionable insights that complement EpiAge-R’s mechanistic scoring [70]. For instance, in the context of partial reprogramming (e.g., OSKM induction), traditional clocks often fluctuate unpredictably. In contrast, EpiAge-R enables precise tracking by detecting the restoration of histone bivalency via the Repair Capacity Index (RCI), distinguishing true rejuvenation from oncogenic dedifferentiation. Similarly, for senolytic therapies targeting SASP, the Physiological Resilience Index (PRI) offers a sensitive readout of systemic inflammatory resolution (e.g., IL-6/IL-10 normalization) that precedes structural tissue changes. By identifying individuals who exhibit high resilience despite chronological ageing, it facilitates the discovery of protective biological pathways [17]. In population studies, resilience-based metrics inform stratification for preventive trials, potentially reducing trial sizes by identifying responders early [10,53].

#### Validation Roadmap: Stress-Recovery Study Design

To empirically validate EpiAge-R as a measure of resilience rather than biological age, we propose a longitudinal “stress-test” study.

- Design: A cohort of older adults undergoing scheduled moderate stress (e.g., elective hip replacement surgery).
- Timeline: Measurements taken at Baseline (T0), Acute Stress (T1: 24h post-op), and Recovery (T2: 30 days post-op).
- Hypothesis: Individuals with a higher pre-operative EpiAge-R score (indicating high resilience) will show a faster return to baseline inflammatory and functional markers at T2, independent of their GrimAge or chronological age. This would demonstrate that EpiAge-R captures the dynamic capacity for recovery missed by static clocks.

## 8. Conclusions

The EpiAge-R framework expands the paradigm of the epigenetic clock from a statistical predictor of chronological age to a mechanistic indicator of biological resilience [15]. By integrating data across molecular (epigenetic and chromatin), physiological (systemic), and environmental layers, EpiAge-R captures not only the pace of ageing but also the system’s capacity for repair, adaptation, and recovery [10]. Through the incorporation of long-read methylation mapping and multi-omics integration, this approach bridges molecular biology with functional geroscience. Ultimately, resilience-based biomarkers derived from EpiAge-R will underpin the design of precision-longevity medicine, guiding strategies that preserve adaptability rather than merely delaying decline [41,47].

## Data Availability

No new data were created or analyzed in this study. Data sharing is not applicable to this article.

## Abbreviations

The following abbreviations are used in this manuscript:

**Abbreviation**	**Definition**
EpiAge-R	Epigenetic Age with Resilience
EDI	Epigenetic Damage Index
RCI	Repair Capacity Index
PRI	Physiological Resilience Index
BAI	Behavioural/Environmental Adaptation Index
CpG	Cytosine-phosphate-Guanine
HRV	Heart-Rate Variability
SASP	Senescence-Associated Secretory Phenotype
LADs	Lamina-Associated Domains
TADs	Topologically Associating Domains
LINC	Linker of Nucleoskeleton and Cytoskeleton
mtDNA	Mitochondrial DNA
SIRT1	Sirtuin 1
BDNF	Brain-Derived Neurotrophic Factor
OXTR	Oxytocin Receptor
HPA	Hypothalamic-Pituitary-Adrenal
HATs	Histone Acetyltransferases
HDACs	Histone Deacetylases
SNPs	Single Nucleotide Polymorphisms
mQTLs	Methylation Quantitative Trait Loci
